# Surface electromyographic characteristics of lower limb muscles in frail older adults: Protocol for an observational case - control study

**DOI:** 10.1371/journal.pone.0325356

**Published:** 2025-07-03

**Authors:** Pingping Huang, Linjing Wu, Shiqi Chen, Jiahua Li, Yu Zhang, Yuan Chen

**Affiliations:** 1 Department of Nursing, Xiamen Cardiovascular Hospital of Xiamen University, School of Medcine, Xiamen University, Xiamen, China; 2 School of Nursing, Fujian University of Traditional Chinese Medicine, Fuzhou, China; PLOS: Public Library of Science, UNITED KINGDOM OF GREAT BRITAIN AND NORTHERN IRELAND

## Abstract

**Background:**

Frailty is a dynamic and reversible clinical syndrome in older adults that profoundly affects an individual’s quality of life, health outcomes, and overall prognosis, thereby underscoring the importance of early identification and timely intervention. Alterations in the biomechanical parameters of lower limb muscles may constitute the pathophysiological underpinnings of frailty. Surface electromyography (sEMG) is a vital tool for assessing neuromuscular function. To date, there are no reports on the characteristics of surface electromyographic in the lower limbs of frail older individuals.

**Methods:**

This is an observational category matching case-control study. We aim to recruit 75 frail older adults and 75 matched non-frail older participants (1:1), to be assigned respectively to the case and control groups. All subjects will undergo sEMG of the lower limb muscles during a 10-meter walk test and will be assessed for gait and balance. The primary outcome will be the difference in sEMG parameters between the two groups, including root mean square (RMS), integrated electromyography (IEMG), averaged electromyography (AEMG), mean power frequency (MPF), and median frequency (MF), as well as those computed from these metrics, including symmetry index (SI), muscle activation sequences, and muscle contribution rates. Secondary outcomes will encompass the time taken to complete the 10-meter walk test and assessments of gait and balance. Furthermore, the relationship between electromyographic parameters and the state of frailty will be evaluated, and the predictive capability of these sEMG parameters for identifying frailty will be explored.

**Trial registration number:**

ChiCTR2300078953

## Introduction

As global aging dynamics intensify, frailty has emerged as a major challenge to healthy aging. Frailty is a clinical syndrome of increased vulnerability and dysfunction due to the cumulative decline of multiple body systems, characterized by unconscious weight loss, reduced grip strength, slowed gait, decreased physical activity and self-reported fatigue, which significantly increases the risk of adverse health outcomes such as falls, disability and death [1–3], and imposes a serious economic burden on the families of patients, making social security for old-age and health care more challenging [1,4,5]. However, the process of frailty can be delayed and reversed, and effective interventions can prevent the occurrence of adverse events in older adults in the pre-frailty stage and delay the entry into the frailty stage, or even recover from the pre-frailty stage to a non-frailty state [1,6], so early screening and identification of high-risk groups is an important part of the optimal management of frailty in older adults.

Slowed gait speed is a component of the frailty phenotype, and studies have shown significant decreases in gait speed in frail older adults, whether they are walking at habitual speeds or walking at fast speeds [7–9]. While gait speed can predict frailty and some adverse health outcomes [10–12], numerous factors in daily life can slow walking speed in older adults, making it a less effective predictor when considered in isolation. Decrements in walking ability may be related to reductions in muscle mass, muscle contractility, balance, and other physiological factors [13–15], with decreased strength and endurance in lower limb muscles strongly correlated with heightened frailty risk [16]. Notably, the rectus femoris, semitendinosus, tibialis anterior, and medial head of gastrocnemius are critical to walking; reductions in their strength may be pivotal to the observed deceleration in gait speed [17,18]. These observations suggest that changes in the biomechanical parameters of lower limb muscles could underpin the pathophysiology of frailty, and identifying these characteristic changes is crucial for developing effective frailty screening, prevention, and rehabilitation programs.

Among the various methods used to assess muscle behavior, electromyography (EMG) is regarded as the ‘gold standard’. Surface electromyography (sEMG), a crucial tool for neuromuscular function research, provides real-time, objective, non-invasive, and dynamically sensitive measurements. It allows for precise monitoring of muscle activities by recording bioelectrical signals from muscles during activity, accurately reflecting changes in muscle activation patterns, fatigue levels, muscle strength, muscle contribution rates, and muscle coordination [19,20]. Although widely used in sports science, sports medicine, and rehabilitation [19,21], there is yet to be research linking sEMG with frailty in older adults.

### Primary and secondary objectives

The primary objective of this study is to compare the surface electromyography (sEMG) signal parameters of the rectus femoris, semitendinosus, tibialis anterior, and medial head of the gastrocnemius muscles in both lower extremities during a 10-meter walk test between frail and non-frail older adults in order to characterize changes in lower extremity sEMG signals in frail older adults.

Secondary objectives are to explore the correlations between EMG parameters and frailty states and to assess the predictive validity and diagnostic value of EMG parameters for frailty states.

## Methods and analysis

### Research design

This is an observational category matching case-control study. The study research will be developed according to the Strengthening the Reporting of Observational Studies in Epidemiology (STROBE) [22] and the Standard Protocol Items: Recommendations for Interventional Trials (SPIRIT) [23] (S1 Appendix).

### Ethics and dissemination

The study will be conducted according to the guidelines of the Declaration of Helsinki and has been approved by the Ethics Committee of Xiamen Cardiovascular Hospital of Xiamen University (Ethical Approval No. [2023] Medical Ethics Science No. 39; approval date: November 8, 2023). Before participation, research assistants will provide a comprehensive explanation of the study’s objectives, methods, potential risks, and benefits, followed by obtaining written informed consent from each participant. Participants will have the right to withdraw at any time without affecting their medical care. All relevant data from the research process will be accurately recorded in the Case Report Forms designed by the research team for subsequent organization and analysis (S2 Appendix).

Access to participant data will be limited to authorized team members only, and all personal identifiers will be removed. Participants will be assigned unique identifiers to ensure confidentiality. The collected data will be securely stored and used exclusively for the purposes defined in the study protocol.

The dissemination of the results will be conducted irrespective of the outcome. Findings will be submitted for publication in peer-reviewed journals and presented at relevant scientific conferences. Summaries of the results will also be made available to participants upon request. All publications and presentations will be based on data that has been fully anonymized to protect participant privacy.

### Study status and timeline

**Fig 1:**
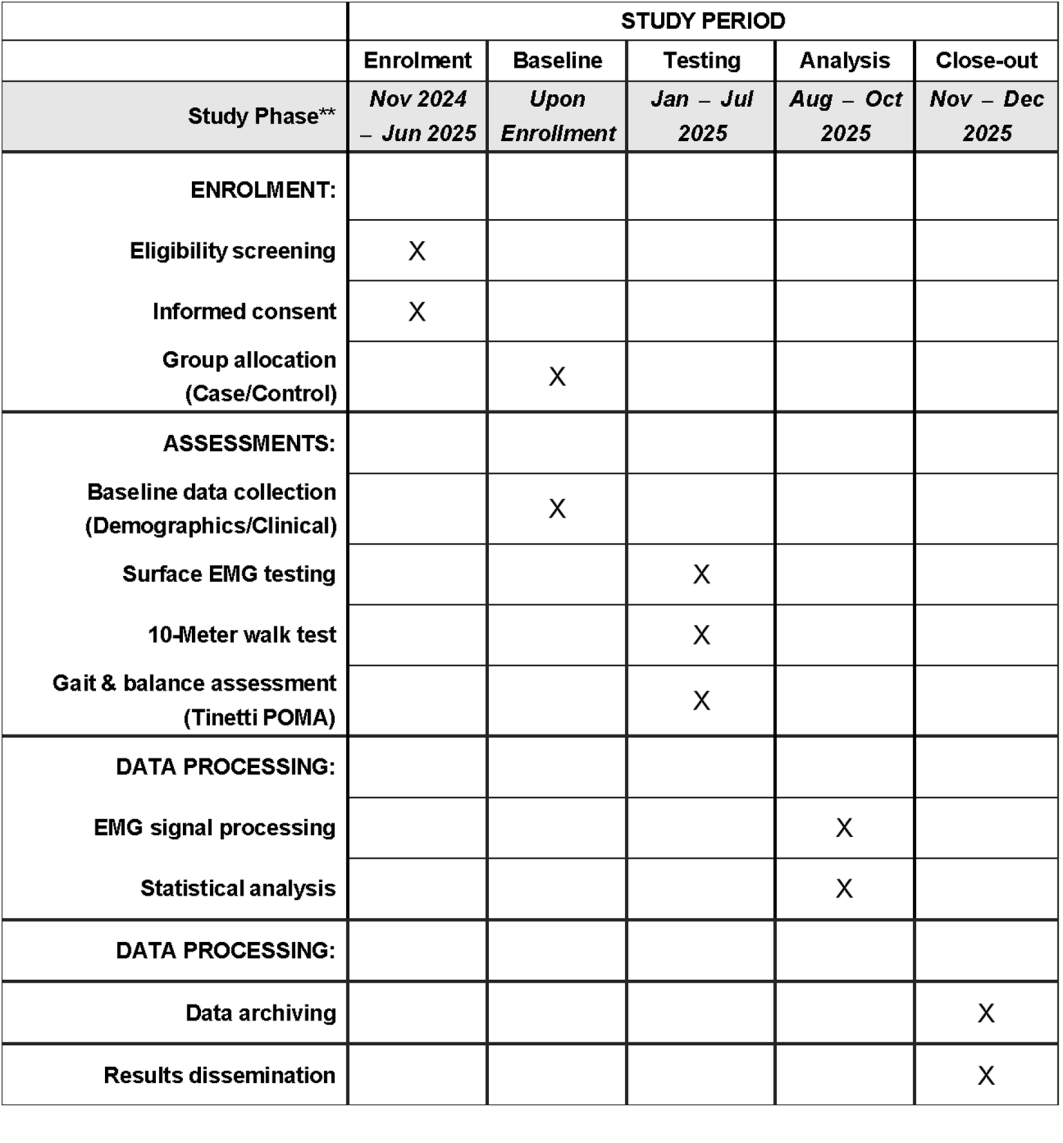
SPIRIT schedule of enrolment, assessments, and analysis phases for the observational case-control study on lower limb sEMG.

This study is currently ongoing. Participant recruitment began in November 2024 and is expected to continue until June 2025. Data collection has been conducted concurrently since January 2025 and is anticipated to conclude in July 2025. Following the completion of data collection, data analysis will be conducted, with an estimated completion date of October 2025. The results of this study are expected to be available shortly thereafter. The timeline presented herein accurately reflects the status of the study as of the manuscript submission date. The study timeline and assessment schedule are detailed in Fig 1.

### Participants

Recruitment for the study will take place from November 2024 to April 2025 using non-randomized convenience sampling. Seventy-five older adults from the inpatient and outpatient departments of the Xiamen Cardiovascular Hospital of Xiamen University in China, who are assessed as frail using the Fried’s frailty phenotype [24], will be selected for the case group. An additional 75 non-frail older adults, matched by age, gender, and cultural background during the same period, will form the control group. All participants will sign an informed consent form (S3 Appendix) and undergo baseline assessments, which include comprehensive socio-demographic and clinical data collection, along with screening using the Fried’s frailty phenotype (S4 Appendix). A panel investigator will review the inclusion and exclusion criteria and assign participants to either the case or control group based on the Fried’s frailty phenotype screening results.

### Selection criteria

#### Inclusion criteria.

(1) Case group
  1) Older individuals evaluated as frail using the Fried’s frailty phenotype [24], which includes five criteria: (i) Unintentional weight loss: a loss of more than 4.5 kg or more than 5% of body weight over the past year, excluding dieting and exercising. (ii) Slowed walking speed: time taken to walk 4.57 meters, assessed in conjunction with height and gender. (iii) Reduced grip strength: older adults are instructed to measure grip strength twice with their usual hand, and the average of these two measurements is evaluated against gender and body mass index (BMI). (iv) Decreased physical activity: measured using the Minnesota Leisure Time Activity questionnaire, with activity levels less than 383 kcal/week for men (approximately 2.5 hours of walking) and less than 270 kcal/week for women (about 2 hours of walking) indicating a reduction. (v) Self-reported fatigue: assessed using two questions from the Center for Epidemiological Studies-Depression (CES-D). Question 1: ‘I felt that everything I did was an effort during the past week.’ Question 2: ‘I could not get “going” during the past week.’ Scoring for questions 1 and 2 assigns 0 points for less than 1 day, 1 point for 1–2 days, 2 points for 3–4 days, and 3 points for more than 4 days, with a score of 2–3 points on any question indicating significant fatigue. One point is awarded for each criterion met, with a higher score indicating greater frailty. Scores of 3 or more indicate frailty, scores of 1–2 indicate pre-frailty, and a score of 0 indicates no frailty. 2) Age 60 years old and above. 3) Those who can walk independently without assistive devices. 4) Those who possess normal reading, understanding, and expressive abilities to engage in relevant assessments and tests. 5) Those who voluntarily participate in the study and sign the informed consent form.
(2) Control group.

The control group will include older adults without frailty, with all other inclusion criteria identical to those of the case group.

### Exclusion criteria: all participants

(1) Complicated with other serious neuromusculoskeletal injuries or diseases.
(2) Complicated with severe cardiovascular, cerebrovascular, or psychiatric conditions.
(3) Inability to perform the required maneuvers.
(4) Allergic to surface electrodes.
(5) Complicated with other serious medical illnesses or other reasons for not being able to complete the test.

### Sample size calculation

The sample size calculation was performed using G*Power 3.1.9.7, based on an effect size of 0.433 derived from pilot study data. With a power of 80% and an alpha level of 0.05, the required sample size was calculated to be 67 subjects per group. Anticipating a 10% attrition rate, the sample size was adjusted to 75 subjects per group, resulting in a total of 150 subjects to ensure adequate power for detecting significant differences.

### Experimental procedures

Eligible participants will receive a detailed explanation of the test methods and procedures. Upon obtaining their informed consent and basic personal data, they will undergo a normal-speed 10-meter walk test and surface electromyographic measurement of lower limb muscles in an EMG room maintained at 25°C.

### Muscles to be tested

Based on the relevant literature and preliminary data analysis, the muscles selected for testing in this study are the rectus femoris, semitendinosus, tibialis anterior, and the medial head of the gastrocnemius in both lower limbs of older adult participants.

### Test program

**Fig 2:**
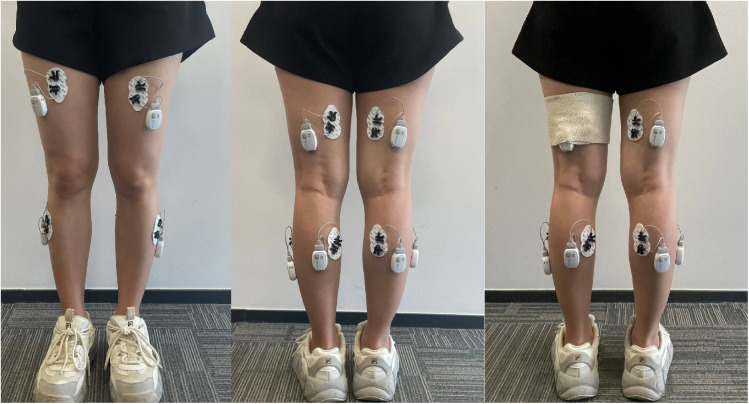
Figure of electrode placement and bandage fixation for surface electromyography recording.

(1) Instrument preparation and electrode placement: Surface EMG signals will be collected using the Dinuo Surface EMG Analyzer and Feedback Instrument (Zhejiang Dinuo Medical Science and Technology Co., Ltd., model SG-800A). Before testing, the instrument is adjusted and set to a sampling frequency of 2000 Hz. This wireless EMG testing method, in contrast to wired electrodes, offers greater convenience and less interference with actions, suitable for a broader range of application scenarios. Participants will be required to expose the skin of their lower limbs, depilate any excessive hair on the muscle surface, and disinfect and degrease the designated testing area using a 75% alcohol cotton ball. Electrodes are placed once the skin has dried to minimize impedance. Placement of all recording electrodes adheres to the European recommendations of the Surface ElectroMyoGraphy for the Non-Invasive Assessment of Muscles (SENIAM). Single-use EMG recording electrode patches (Zhejiang Dinuo Medical Technology Co., Ltd., model E5) are affixed to the rectus femoris (midpoint between the anterior superior iliac spine and the patella’s upper edge), semitendinosus (midpoint between the ischial tuberosity and the tibia’s medial epicondyle), tibialis anterior (one-third along the line from the fibula’s tip to the inner ankle’s tip), and medial head of the gastrocnemius (muscle’s most prominent bulge). Electrodes are placed on the muscle belly’s most prominent part, parallel to the muscle fibers. A reference electrode patch is placed on a non-tested muscle to avoid test interference. Electrodes are secured with elastic bandages to prevent displacement and movement during the walk, thereby reducing motion artifacts. The illustration of electrode placement and bandage fixation is shown in Fig 2.

(2) 10-meter walk test: A 12-meter stretch will be marked on an open and flat 15-meter walkway, incorporating a 10-meter timed section flanked by 1-meter zones for acceleration and deceleration at both ends. After the surface EMG recording and reference electrodes have been properly placed, subjects will be instructed to stand still and relaxed at the starting line, with the aim of maintaining the EMG signal near the baseline (<10 μV) as displayed on the instrument. Upon the ‘start’ signal, participants will walk to the finish line at a comfortable pace, maintaining a natural walking speed. The time taken to traverse the 10-meter timed section will be recorded using a timer, and dynamic sEMG parameters of the lower limb muscles will be captured by the surface EMG device, complemented by recordings of different gait cycles using an infrared camera system for further analysis. Typically, three tests will be conducted with 1–2 minutes of rest between each, and the average result of these three tests will be calculated. The entire test will require the accompaniment of a researcher to ensure safety, without any physical contact, and no assistive devices will be used by the subjects during the test.

### Data processing

Surface EMG data analysis will be performed using the proprietary wireless surface EMG analysis feedback software (Zhejiang Dino Medical Technology Co., Ltd.). Data from five gait cycles following the third cycle (i.e., the 4th to 8th gait cycles) will be analyzed. First, the surface EMG signals will be band-pass filtered using a zero-delay Butterworth filter between 20 and 500 Hz to remove noise and irrelevant frequencies from the signals. Afterward, adaptive filtering will be applied to eliminate ECG interference. Once filtering is complete, an analyst will visually inspect the raw EMG data to identify any motion artifacts or noise. The EMG signals will then be rectified and smoothed (using averaging) to convert negative values into positive ones and ensure signal smoothing. However, smoothing filters will not be applied for frequency-domain analysis. To reduce inter-individual variability, the maximum value of the rectified EMG signals (EMG_max) will serve as a benchmark for normalization. Finally, the rectified EMG signals from the 5 gait cycles will be normalized to EMG_max, meaning that all EMG values will be expressed as a ratio relative to EMG_max.

### Outcome measures and calculation algorithms

#### Primary outcome.

The primary outcomes will include three time-domain indicators and two frequency-domain indicators calculated through software analysis, along with the symmetry index (SI), muscle activation sequences, and muscle contribution ratios derived from these indicators.

(1) The time-domain indicators will include root mean square amplitude (RMS), integral electromyography (IEMG), and average electromyography (AEMG), which are primarily related to muscle contraction force. The formulas for these calculations are as follows:

$$\begin{array}{c} RMS\;=\;\sqrt{\frac{\sum {\mathrm{\backslash nolimits}}_{i=0}^{N}Data{\left[i\right]}^{2}}{N}} \end{array}$$

$$\begin{array}{c} IEMG\;=\;\sum_{i=0}^{N}\left|Data[i]\right|\times \Delta t \end{array}$$

$$AEMG=\frac{1}{N}\sum_{i=1}^{N}\left|xi\right|$$

In the aforementioned formulas, Data[i]denotes the EMG signal value at the i-th sampling point. Nrefers to the total number of sampling points, which represents the number of data points collected over a specific time period. iis the index indicating the position of the data point, ranging from the first to the N-th. Δtrepresents the time interval between consecutive sampling points. xi denotes the absolute value of the EMG signal at the i-th sampling point.

(2) The frequency-domain indicators will include mean power frequency (MPF) and median frequency (MF), commonly used to assess muscle fatigue.

$$MPF=\frac{\sum {\mathrm{\backslash nolimits}}_{i=1}^{N}f_{i}\cdot P_{i}}{\sum {\mathrm{\backslash nolimits}}_{i=1}^{N}P_{i}}$$

MF=Median(f(P(f)))

In the aforementioned formulas, $f_{i}$represents the i-th frequency point, while $P_{i}$corresponds to the power spectral density value at the i-th frequency point. Nis the total number of frequency points. P(f)is the power spectral density as a function of frequency f, and frefers to the frequency point.

(3) SI: The formula for calculating SI in this study will be SI = (left RMS – right RMS)/ (left RMS + right RMS). A result greater than 0 will indicate that the discharge from the muscles on the left side is greater than that on the right, and a result less than 0 will indicate the opposite.
(4) Muscle activation sequences: When the average of 10 consecutive sampling points of the EMG signal first exceeds the baseline value (where the baseline is defined as 1.7 times the maximum value of the first 20 sampling points), that time point is recorded as the muscle activation onset time. Subsequently, when the average of 10 consecutive sampling points falls below the baseline, that time point is recorded as the muscle activation offset time. By sorting the activation onset times of each muscle, the muscle that activates earliest is ranked first, determining the muscle activation sequence.
(5) Muscle contribution rate: This rate will be defined as the percentage of the IEMG values of each test muscle relative to the total IEMG values of all test muscles across the five analyzed gait cycles [25]. This measure will reflect the extent of muscle involvement during the walking process.

### Secondary outcome

Secondary outcomes will include the average time of three 10-meter walk tests and assessments of gait and balance abilities. After subjects have completed the walk tests and sufficiently rested, gait and balance will be evaluated using the Tinetti Performance-Oriented Mobility Assessment (Tinetti POMA) (S5 Appendix). This comprehensive tool for assessing gait and balance in older adults was developed by Tinetti in 1986 and later modified by Cobbs et al. in 1999 [26]. The modified version of the Tinetti POMA scale includes a balance test with 9 items totaling 16 points and a gait test with 7 items totaling 12 points; the full scale reaches a maximum of 28 points, with higher scores indicating better balance and walking capabilities, and research has demonstrated that the Tinetti POMA scale possesses good reliability and validity [27]. Assessors receive standardized training prior to evaluations to ensure they can competently demonstrate and instruct each item according to the Tinetti POMA guidelines before conducting assessments.

### Statistical analysis

Statistical analysis will be conducted using SPSS 24.0 software, with graphical representations generated through Graph Pad Prism 8. Measurement data will be analyzed by the Kolmogorov-Smirnov test (K-S test), expressed as mean ± standard deviation (x̅ ± s) if they adhere to a normal distribution, with comparisons between groups made using the two independent samples t-test. For non-normally distributed data, results will be described using the median (interquartile range) [M(IQR)], and comparisons between groups will be conducted using the Mann-Whitney U test. Count data will be expressed as frequency (n) and constitutive ratio (%), with comparisons between groups performed using the χ^2^ test or Fisher’s exact test, and rank data comparisons utilizing the Wilcoxon rank-sum test. Effect sizes will be calculated using Cohen’s d value, and the significance level will be set at a two-sided *P *< 0.05.

The intraclass correlation coefficient (ICC) analysis, specifically the ICC(3,k) model, will be used to quantify the consistency and reproducibility of measurements. To assess the reliability of single measurements, the standard error of measurement (SEM) will be calculated using the formula SEM = SD/$\sqrt{n}$, where n represents the number of measurements per participant (i.e., 3). Additionally, the minimum detectable difference (MDD) will be calculated using the formula MDD = SEM × 1.96×$\sqrt{2}$ to determine whether observed differences exceed those expected from measurement error alone, at the 95% confidence level.

Associations between sEMG parameters and frailty status will initially be assessed using Pearson correlation analysis for normally distributed data or Spearman rank correlation analysis for non-normally distributed data. Subsequently, EMG parameters significantly associated with frailty status will be included in binary logistic regression models to further assess their predictive ability for frailty status, controlling for potential confounders such as age and gender. Finally, EMG parameters that prove significant in the logistic regression analysis will be evaluated for their sensitivity, specificity, and cutoff values for diagnosing frailty using receiver operating characteristic (ROC) curves.

### Quality control

In the present study, comprehensive quality control measures will be implemented to ensure that every stage from participant recruitment to data analysis is performed in strict accordance with standard operating procedures. All potential subjects will undergo a detailed screening process and sign an informed consent form prior to participation. Data collection will be carried out by professionally trained and accredited rehabilitation physicians and research assistants to ensure the accuracy and consistency of the questionnaires and related tests. To prevent data entry errors, a dual data entry system will be employed, and data will be regularly checked for completeness and accuracy. Data hypothesis testing will be conducted prior to statistical analysis to ensure that appropriate statistical methods are used and potential confounding variables are considered in the analysis. The study will adhere to strict ethical guidelines to safeguard participant privacy and data security, and participants will be allowed to withdraw from the study at any time. The entire process will be overseen by an independent quality control team to ensure high standards and reliability of the study. These measures will collectively ensure the validity of the study results and provide a solid scientific basis for understanding the characteristics of EMG signals in frail older adults.

## Discussion

Frailty’s impact on older adults’ health has emerged as a focal point of recent research. Frailty is a dynamic, reversible geriatric syndrome that exists between independence and the need for care. Effective preventive interventions can significantly mitigate its onset and progression [28]. As the population ages, researchers have conducted extensive studies on the measurement of frailty, exploration of its pathogenesis, and the development of management strategies [29]. Despite these efforts, research into the pathogenesis of frailty remains less developed compared to other areas. Notably, no studies have specifically examined the surface electromyography characteristics of the lower extremities in frail older adults. Globally, there is no established gold standard for screening frailty, yet walking speed—a component of the Fried phenotype and the most extensively studied single frailty assessment [24]—has proven to be a crucial independent predictor of adverse clinical outcomes and patient-reported results [30,31]. Walking involves complex systemic mechanisms, and the specifics of its slowdown are not fully understood; however, lower limb biomechanics represent one of the key elements. Moreover, the application of surface electromyography has recently extended beyond traditional fields into areas such as obstetrics, preventive medicine, aging research, and robotics, covering applications in gait and posture analysis, biofeedback, motor synergy, and modeling [19,32]. Thus, exploring the surface EMG characteristics of frail older adults’ lower limbs holds significant clinical importance and could (1) provide sensitive screening indicators for the early detection and prevention of frailty, enabling timely medical intervention and optimization of treatment strategies, (2) offer new insights into the complex mechanisms of age-related frailty, enhancing our biological understanding of this condition, and (3) serve as a scientific basis for assessing lower limb muscle function and developing rehabilitation programs, thereby improving the quality of life for older adults.

Some limitations of this study should be acknowledged. Firstly, as a case-control study, it will not include follow-ups to determine if non-frail older adults who later develop frailty will have surface EMG parameters similar to those observed in the frail older adults; this makes it impossible to definitively establish a causal relationship between changes in surface EMG parameters and frailty. Therefore, we plan to conduct follow-up studies to track the progression of these participants. Secondly, due to financial and time constraints, this study will not be a large-scale multicenter study nor will it analyze a wide range of muscle groups. However, the analysis will strictly adhere to established protocols to ensure as much precision as possible. We aim to include more comprehensive muscle group analyses in future studies.

## Supporting information

S1 Appendix SPIRIT Checklist. (DOC)

S2 Appendix Case report form. (DOCX)

S3 Appendix Informed consent form. (DOCX)

S4 Appendix Fried’s frailty phenotype. (DOCX)

S5 Appendix Tinetti POMA. (DOCX)
