# Supplementary material for: Surface electromyographic characteristics of lower limb muscles in frail older adults: Protocol for an observational case - control study
Source: PLoS One. 2025 Jul 3;20(7):e0325356. doi: 10.1371/journal.pone.0325356 (PMC12225879; doi:10.1371/journal.pone.0325356)
Supplement: S2 Appendix — (DOCX) [file pone.0325356.s002.docx]

| Study ID Number | Research Subject ID  \|_\|_\|_\| | □Completed □Dropped Out |
| --- | --- | --- |
|  |  | □Excluded |

Surface electromyographic characteristics of lower limb muscles in frail older adults

(Case Report Form)

Version: 1.0 Date: 2023-10-16

Name of Study Unit: Xiamen Cardiovascular Hospital of Xiamen University

Investigator Signature:

Subject Name Initials： |_|_|_|_|

Informed Consent Date: Day _ _ Month _ _ 202_

Enrollment Date: Day _ _ Month _ _ 202_

Study End Date: Day _ _ Month _ _ 202_

**Instructions for Completing the Case Report Form**

1.The case report form should be completed in pen with clear and legible handwriting.

2.It is essential to complete the form in a timely, accurate, clear, and comprehensive manner. Corrections must be made by striking through errors with a horizontal line, along with the initials and date of correction. Example: ~~58.6~~ 56.8 ^LGW 12.02.12^.

3.The initials section for the participant's name should be fully filled. For a two-character name, fill in the initials of both characters; for a three-character name, use the initials of the first three characters and the second letter of the third character; for a four-character name, use the first letter of each character.

4.All option boxes must be marked with an “×”. All fields in the tables must be filled with the appropriate text or number.

5.Laboratory test reports should be pasted in the designated area (report attachment area). If the original report cannot be retained, the results must be copied accurately in the designated area (report attachment area).

6.During the trial, adverse event records should be filled in truthfully, including the occurrence time, severity, duration, measures taken, and outcomes. For any severe adverse events (including hospitalization, extended hospital stays, disability, work incapacity, life-threatening conditions, death, congenital malformations, etc.), the Research Department and Hospital Ethics Committee must be notified immediately.

7.The clinical trial should be conducted strictly in accordance with the study protocol. The checks and records to be completed during each phase of the study should be carried out in accordance with the clinical research workflow chart.

**Work flow chart of “Surface electromyographic characteristics of lower limb muscles in frail older adults”**

| **Time**  **Project** | **Screening Period/**  **Baseline** | **After 10-meter Walk Test** |
| --- | --- | --- |
| **Collect basic information** |  |  |
| Sign informed consent form | × |  |
| Collect baseline data | × |  |
| Review of inclusion and exclusion criteria | × |  |
| **Primary outcomes** |  |  |
| Root mean square amplitude (RMS) |  | × |
| Integral electromyography (IEMG) |  | × |
| Average electromyography (AEMG) |  | × |
| Mean power frequency (MPF) |  | × |
| Median frequency (MF) |  | × |
| Symmetry index (SI) |  | × |
| Muscle activation sequence |  | × |
| Muscle activation duration |  | × |
| Muscle contribution ratio |  | × |
| **Secondary outcomes** |  |  |
| Time of 10-meter walk test |  | × |
| Gait and balance ability |  | × |
| **Other tasks** |  |  |
| Recording of adverse events | × | × |
| Study completion report |  | × |

| Research Subject ID | Screening Date | Screening/Baseline  Case Enrollment |
| --- | --- | --- |
| \|_\|_\|_\| | Day _ _ Month _ _ 202_ |  |

**1. Inclusion and exclusion criteria**

| **Inclusion criteria:** | **Yes** | **No** |
| --- | --- | --- |
| 1. Age 60 years old and above. | □ | □ |
| 1. Those who can walk independently without assistive devices. | □ | □ |
| 1. Those who possess normal reading, understanding, and expressive abilities to engage in relevant assessments and tests. | □ | □ |
| 1. Those who voluntarily participate in the study and sign the informed consent form. | □ | □ |
| **If the answer to any of the above is "No", the subject cannot participate in the trial** | | |

| **Exclusion criteria:** | **Yes** | **No** |
| --- | --- | --- |
| (1) Complicated with other serious neuromusculoskeletal injuries or diseases. | □ | □ |
| (2) Complicated with severe cardiovascular, cerebrovascular, or psychiatric conditions. | □ | □ |
| (3) Inability to perform the required maneuvers. | □ | □ |
| (4) Allergic to surface electrodes. | □ | □ |
| (5) Complicated with other serious medical illnesses or other reasons for not being able to complete the test. | □ | □ |
| **If the answer to any of the above is "yes", the subject cannot participate in the trial** | | |

Investigator: Record Date:

| Research Subject ID | Screening/Baseline  Case Enrollment |
| --- | --- |
| \|_\|_\|_\| |  |

Date of admission:

| Baseline data |
| --- |
| Name pinyin abbreviation:\| \| \| \| \|  Gender: □Male □Female  Age: __________  Height: __________m  Weight: __________kg  Body Mass Index (BMI): __________  Education level: □Junior high school and below □High school/Technical secondary school □Junior college/Undergraduate □Graduate and above  Marital status: □Unmarried □Married □Divorced □Widowed  Living style: □Share living with others □Living alone  Medication: □No medication □≤ 4 species/day □≥ 5 species/day  Medical History: □Diabetes mellitus □Hypertension □Hyperlipidemia □Other:_____□None  History of muscle or joint injury: □Yes, specific circumstances:___________ □None  Dietary preference: □Vegetarian □Meat food □Meat and vegetable combination □Staple food  Whether you have smoking habits: □Yes (smoke at least one cigarette a day for more than half a year), frequency and amount:__________ □None  Whether you have drinking habits: □Yes (drink at least once a week for more than half a year), frequency and amount:__________ □None  Whether there is physical activity and exercise: □Yes (at least three times a week, each time for 30 minutes or more), frequency and time:__________ □None  Health self-assessment: □Very good □Good □General □Poor □Very poor |
| **Frail □None □Yes, fill in the details of the Frailty Phenotype (FP) scoring situation** |
| Unintentional weight loss: □ 0 points □ 1 point  Slowed walking speed: □ 0 points □ 1 point  Decreased grip strength: □ 0 points □ 1 point  Reduced physical activity: □ 0 points □ 1 point  Self-reported exhaustion: □ 0 points □ 1 point |

Investigator: Record Date:

| Research Subject ID | After 10-meter walking test |
| --- | --- |
| \|_\|_\|_\| |  |

| **Surface electromyography parameters** | **Tested Muscle** | **Test Date** | **Test Value** |
| --- | --- | --- | --- |
| Root mean square amplitude (RMS) | Left rectus femoris |  |  |
|  | Right rectus femoris |  |  |
|  | Left semitendinosus |  |  |
|  | Right semitendinosus |  |  |
|  | Left tibialis anterior |  |  |
|  | Right tibialis anterior |  |  |
|  | Left medial gastrocnemius |  |  |
|  | Right medial gastrocnemius |  |  |
| Integral electromyography (IEMG) | Left rectus femoris |  |  |
|  | Right rectus femoris |  |  |
|  | Left semitendinosus |  |  |
|  | Right semitendinosus |  |  |
|  | Left tibialis anterior |  |  |
|  | Right tibialis anterior |  |  |
|  | Left medial gastrocnemius |  |  |
|  | Right medial gastrocnemius |  |  |
| Average electromyography (AEMG) | Left rectus femoris |  |  |
|  | Right rectus femoris |  |  |
|  | Left semitendinosus |  |  |
|  | Right semitendinosus |  |  |
|  | Left tibialis anterior |  |  |
|  | Right tibialis anterior |  |  |
|  | Left medial gastrocnemius |  |  |
|  | Right medial gastrocnemius |  |  |
| Mean power frequency (MPF) | Left rectus femoris |  |  |
|  | Right rectus femoris |  |  |
|  | Left semitendinosus |  |  |
|  | Right semitendinosus |  |  |
|  | Left tibialis anterior |  |  |
|  | Right tibialis anterior |  |  |
|  | Left medial gastrocnemius |  |  |
|  | Right medial gastrocnemius |  |  |
| Median frequency (MF) | Left rectus femoris |  |  |
|  | Right rectus femoris |  |  |
|  | Left semitendinosus |  |  |
|  | Right semitendinosus |  |  |
|  | Left tibialis anterior |  |  |
|  | Right tibialis anterior |  |  |
|  | Left medial gastrocnemius |  |  |
|  | Right medial gastrocnemius |  |  |
| Symmetry index (SI) | Left rectus femoris |  |  |
|  | Right rectus femoris |  |  |
|  | Left semitendinosus |  |  |
|  | Right semitendinosus |  |  |
|  | Left tibialis anterior |  |  |
|  | Right tibialis anterior |  |  |
|  | Left medial gastrocnemius |  |  |
|  | Right medial gastrocnemius |  |  |
| Muscle activation sequences | Left rectus femoris |  |  |
|  | Right rectus femoris |  |  |
|  | Left semitendinosus |  |  |
|  | Right semitendinosus |  |  |
|  | Left tibialis anterior |  |  |
|  | Right tibialis anterior |  |  |
|  | Left medial gastrocnemius |  |  |
|  | Right medial gastrocnemius |  |  |
| Muscle activation durations | Left rectus femoris |  |  |
|  | Right rectus femoris |  |  |
|  | Left semitendinosus |  |  |
|  | Right semitendinosus |  |  |
|  | Left tibialis anterior |  |  |
|  | Right tibialis anterior |  |  |
|  | Left medial gastrocnemius |  |  |
|  | Right medial gastrocnemius |  |  |
| Muscle contribution ratios | Left rectus femoris |  |  |
|  | Right rectus femoris |  |  |
|  | Left semitendinosus |  |  |
|  | Right semitendinosus |  |  |
|  | Left tibialis anterior |  |  |
|  | Right tibialis anterior |  |  |
|  | Left medial gastrocnemius |  |  |
|  | Right medial gastrocnemius |  |  |
| **Secondary Indicators** |  | **Test Date** | **Test Value** |
| 10-meter walking time |  |  |  |
| Gait and balance ability |  |  |  |

Investigator: Record Date:

| Research Subject ID | Adverse Event Record Date | Adverse Event Form |
| --- | --- | --- |
| \|_\|_\|_\| | Day _ _ Month _ _ 202_ |  |

| Adverse Event: □ Yes □ No (If "Yes," please continue filling out the form) | | | |
| --- | --- | --- | --- |
| Regardless of whether it is related to the trial, all adverse events should be recorded. | | | |
| Name of adverse event |  |  |  |
| Start date  (Day/Month/Year) |  |  |  |
| End date  (Day/Month/Year) |  |  |  |
| Severity of Adverse Event | □ Mild □ Moderate □ Heavy | □ Mild □ Moderate □ Heavy | □ Mild □ Moderate □ Heavy |
| Outcome of adverse events | - Vanish - Remission - Continue - Don't know - Death | - Vanish - Remission - Continue - Don't know - Death | - Vanish - Remission - Continue - Don't know - Death |
| Relationship to this study | - Definitely related - Most likely to be related to - Possibly related to - Possibly irrelevant - To be evaluated - Undeterminable - Have nothing to do with | - Definitely related - Most likely to be related to - Possibly related to - Possibly irrelevant - To be evaluated - Undeterminable - Have nothing to do with | - Definitely related - Most likely to be related to - Possibly related to - Possibly irrelevant - To be evaluated - Undeterminable - Have nothing to do with |
| Measures taken | - Withdrawal from study - Treatment administered - No action taken - Others | - Withdrawal from study - Treatment administered - No action taken - Others | - Withdrawal from study - Treatment administered - No action taken - Others |

Investigator: Record Date:

| Research Subject ID | Completion Date | Trial Completion Form |
| --- | --- | --- |
| \|_\|_\|_\| | Day _ _ Month _ _ 202_ |  |

| Trial Completion Form |
| --- |
| Informed consent signing date: Day _ _ Month _ _ 202_  Date of enrollment: Day _ _ Month _ _ 202_ |
| Did any adverse events occur during the trial period? □ Yes □ No  If "Yes," have the adverse events been resolved? □ Yes □ No, if "No," monitoring of adverse reactions should continue until stability or resolution. |
| - Did the participant complete the study? □ Yes □ No - Main reason for terminating the trial: (Select one) - □ Adverse event (Fill in the adverse event form) - □ Lost to follow-up (Including automatic withdrawal by the participant) - □ Withdrawn by the investigator - □ Other: ______ |

Investigator: Record Date:

Report paste place

|  |
| --- |

Principal Investigator Review Statement

As the person responsible for this center, I hereby declare:

I have reviewed all the contents and data in this case report form, confirmed that the recorded information is true and accurate, that all items are fully completed, and that all errors or omissions have been corrected or annotated. I hereby declare that it meets the requirements of the trial plan.

Principal Investigator's Signature:

Day _ _ Month _ _ 202 _ _
