# Supplementary material for: Surface electromyographic characteristics of lower limb muscles in frail older adults: Protocol for an observational case - control study
Source: PLoS One. 2025 Jul 3;20(7):e0325356. doi: 10.1371/journal.pone.0325356 (PMC12225879; doi:10.1371/journal.pone.0325356)
Supplement: S3 Appendix — (DOCX) [file pone.0325356.s003.docx]

**Surface electromyographic characteristics of lower limb muscles in frail older adults**

**Informed Consent Form**

Name of research program: Surface electromyographic characteristics of lower limb muscles in frail elderly

Protocol version number: V1.0 Date: October 16, 2023

Informed consent version number: V1.0 Date: October 16, 2023

Research institution: Xiamen Cardiovascular Hospital of Xiamen University

Principal investigator: Yuan Chen

Dear Participant,

You are invited to participate in a research study titled "Surface electromyographic characteristics of lower limb muscles in frail older adults" approved and to be conducted at the Xiamen Cardiovascular Hospital of Xiamen University. Approximately 150 volunteers are expected to participate in this study, which has received ethical review and approval from the Ethics Committee of Xiamen Cardiovascular Hospital of Xiamen University.

**1. Why is this study being conducted?**

As the population ages, frailty becomes a significant challenge and a major health issue for the elderly. Early identification and intervention are crucial for delaying or reversing frailty. Changes in the biomechanics of lower limb muscles are a pathological basis of frailty, and surface electromyography (sEMG) can provide real-time data reflecting the activity and functional status of muscles. Characteristics of lower limb muscle sEMG may become valuable predictive and screening markers for elderly frailty. Therefore, this project aims to study changes in the sEMG signals of lower limb muscles in frail elderly individuals to provide references for early identification and prevention of frailty and its adverse events.

**2. How many people will participate in this study?**

The Xiamen Cardiovascular Hospital of Xiamen University plans to invite 150 people to participate in this research.

**3. How long will the study last?**

The study will last for 14 months, from November 2024 to December 2025. You may choose to withdraw from the study at any time without losing any benefits you are entitled to. If you decide to withdraw during the study, we encourage you to discuss this with the researchers.

**4. What does the study involve?**

If you agree to participate, please sign this informed consent form.

The study will measure the sEMG signals of the rectus femoris, semitendinosus, tibialis anterior, and medial head of the gastrocnemius muscles in frail and non-frail elderly individuals during a 10-meter walk test. The aim is to compare and analyze signal parameters between the two groups to clarify the changes in sEMG signals in the lower limbs of frail elderly individuals.

**5. What treatments will I receive if I participate in the study?**

If you agree to participate in this study, it will not affect any of your usual treatments. You will be required to assist with the collection of surface EMG signals from the rectus femoris, semitendinosus, tibialis anterior, and medial head of the gastrocnemius muscles of both lower limbs during a 10-meter walk test. This test is non-invasive, painless, and harmless, and it will provide information about your lower limb strength, coordination, and balance. Additionally, we will collect your age, gender, educational level, marital status, living situation, smoking and drinking habits, medication use, and dietary preferences. All tests and assessments are free of charge. (If you choose not to participate in this study, you will not need to undergo the lower limb surface electromyography test or share the aforementioned personal information.)

**6. Do I have other treatment options?**

This study does not involve any treatment, and your participation or non-participation will not affect any treatment you are entitled to receive.

**7. Who can participate in this study?**

Participants eligible for this study are: (1) Aged 60 years and above; (2) Able to walk independently without assistance; (3) Capable of reading, understanding, and expressing themselves adequately to undergo the relevant assessments and tests; (4) Willing to participate in this study and sign the informed consent form. The final decision on your participation will be made after assessment by professionals.

**8. Who should not participate in this study?**

If you have other serious neuromusculoskeletal injuries/diseases, severe cardiovascular and cerebrovascular diseases or psychiatric disorders, are unable to perform the required study activities, or are allergic to surface electrodes, you are not eligible for this study.

**9. What are the possible adverse reactions, risks, and inconveniences of participating in the study?**

Participation in this study will not cause you any adverse reactions or risks. The study will require some of your time, which may cause inconvenience or trouble.

**10. What are the benefits of participating in the study?**

By participating in this study, your health will be closely monitored by the researchers during the study period. The surface electromyographic indicators collected and the predictions based on the data may provide references for subsequent treatment and care, potentially improving your health.

**11. Will I need to pay any fees to participate in the study?**

There are no additional costs for participating in this study. This study does not affect any of your existing treatments, the costs of which you will still need to bear. The costs associated with surface electromyography testing and risk factor assessment related to the study will be covered by the research team.

**12. What happens if I am injured during the study?**

Participation in this study is not expected to cause any physical harm. Even if you have signed this informed consent form, you still retain all your legal rights.

**13. Will my personal information be kept confidential?**

Your medical records will be kept at the hospital, and access to your records will be allowed for researchers, regulatory authorities, and the ethics committee. Any public reports about the results of this study will not disclose your personal identity. We will make every effort within the bounds of the law to protect the privacy of your personal medical information.

**14. Must I participate in the study?**

Participation in this study is entirely voluntary. You may refuse to participate or withdraw from the study at any time without any reason. This decision will not affect the treatment your doctor provides you.

**15. How will participating in the study affect my life?**

You may find the tests and assessments inconvenient and require special arrangements.

**16. Where can I seek advice?**

If you have any questions related to this study, please contact Pingping Huang at fixed phone: 0592-2993237 or mobile: 13509388294.

If you have any questions related to your rights, or if you wish to express dissatisfaction or concerns during the participation in the study, please contact the Ethics Committee Office at phone: 0592-2292562.

**Informed Consent Signature Page**

I have read this informed consent form.

I had the opportunity to ask questions, and all my questions have been answered.

I understand that participation in this study is voluntary.

I can choose not to participate in this study, or to notify the researcher at any time that I wish to withdraw without any discrimination or retaliation, and my medical treatment and rights will not be affected.

If I require other treatments, or if I do not follow the study protocol, or if an injury related to the study occurs, or for any other reason, the study physician may terminate my continued participation in this study.

I will receive a signed copy of this “Informed Consent Form”.

Participant's Name (Print): __________________

Participant's Signature: _____________________Contact Information: _________________________

Date: __________________

Legal Representative's Name (Print): ______________

Legal Representative's Signature: _________________Contact Information: _____________________

Date: __________________

I certify that all information and any other written information were accurately explained to the participant or their guardian, and the participant or legal representative has fully understood this information. I further certify that the participant (or by their legal representative) has voluntarily agreed to participate in this study.

Impartial Witness Name (Print): ________________ID Number:_________________________

Impartial Witness Signature:____________________Contact Information: _________________

Date: __________________

I have accurately informed the participant, he/she has read this informed consent form accurately, and had the opportunity to ask questions.

Researcher's Name:________________________

Researcher's Signature: ____________________Contact Information:______________________

Date: __________________

(*Note: If the participant is illiterate, a witness's signature is still required; if the participant is incapable of acting, then a legal representative's signature is required.*)
