# Supplementary material for: Surface electromyographic characteristics of lower limb muscles in frail older adults: Protocol for an observational case - control study
Source: PLoS One. 2025 Jul 3;20(7):e0325356. doi: 10.1371/journal.pone.0325356 (PMC12225879; doi:10.1371/journal.pone.0325356)
Supplement: S4 Appendix — (DOCX) [file pone.0325356.s004.docx]

**Frailty Phenotype Scale (FP)**

Instructions: For the following questions, please assess whether you have experienced frailty. There are 5 questions in total. Please mark "✓" in the box that corresponds to your actual situation.

| **Evaluation Indicator** | **Male** | **Female** |
| --- | --- | --- |
| Unintentional Weight Loss | Height: ____ cm; Weight: ____ kg; BMI: ____  Over the past year, weight loss > 4.5 kg (excluding dieting and exercise) or > 5% of body weight.  □Yes  □No | |
| Slowed Walking Speed | Measure the time required to walk 4.57 m. Evaluate based on height and gender. | |
|  | Walking Time: ______  □Height ≤ 173 cm, ≥ 7 sec  □Height > 173 cm, ≥ 6 sec | Walking Time: ______  □Height ≤ 159 cm, ≥ 7 sec  □Height > 159 cm, ≥ 6 sec |
| Weak Grip Strength | Measurement Method: Use a dynamometer to measure grip strength while seated, with both feet resting naturally on the floor, knees at 90 degrees, shoulder adducted to neutral position, elbow at 90 degrees, forearm in neutral position, and wrist at 0-30 degrees extension. Instruct the elderly to perform two measurements for each hand, and use the average of all measurements. | |
|  | Grip Strength Value:  □BMI≤24，≤29kg  □24＜BMI≤26，≤30kg  □26＜BMI≤28，≤30kg  □BMI＞28，≤32kg | Grip Strength Value:  □BMI≤23，≤17kg  □23＜BMI≤26，≤17.3kg  □26＜BMI≤29，≤18kg  □BMI＞29，≤21kg |
| Reduced Physical Activity | Assessed using the Minnesota Leisure Time Physical Activity Questionnaire (MLTA), based on estimated weekly caloric expenditure during physical activity. | |
|  | ＜< 383 kcal/week (approximately equivalent to 2.5 hours of walking) | ＜< 270 kcal/week (approximately equivalent to 2 hours of walking) |
| Self-Reported Exhaustion | Assessed using 2 items from the Center for Epidemiologic Studies Depression Scale (CES-D). Any item rated 2-3 points is considered positive.   1. "During the past week, I felt that everything I did was an effort."   □≤ 1 day (0 points)  □1-2 days (1 point)  □3-4 days (2 points)  □> 4 days (3 points)   1. "During the past week, I could not get going."   □≤ 1 day (0 points)  □1-2 days (1 point)  □3-4 days (2 points)  □> 4 days (3 points) | |
| Scoring Criteria: Each evaluation indicator: "Yes" = 1 point, "No" = 0 points.  0 points indicates robust health; 1-2 points indicate pre-frailty; 3 or more points indicate frailty. | | |
