# Supplementary material for: Surface electromyographic characteristics of lower limb muscles in frail older adults: Protocol for an observational case - control study
Source: PLoS One. 2025 Jul 3;20(7):e0325356. doi: 10.1371/journal.pone.0325356 (PMC12225879; doi:10.1371/journal.pone.0325356)
Supplement: S5 Appendix — (DOCX) [file pone.0325356.s005.docx]

**Tinetti Performance-Oriented Mobility Assessment (Tinetti POMA)**

| **Part One: Balance Test**  (Patient sits on an armless chair) | | |
| --- | --- | --- |
| **Evaluation Item** | **Evaluation Criteria** | **Score** |
| 1. Sitting Balance | Tilting or sliding in chair = 0 |  |
|  | Able to sit safely = 1 |  |
| 2. Arising | Unable without assistance = 0 |  |
|  | Able but requires armrest = 1 |  |
|  | Able without armrest = 2 |  |
| 3. Attempting to Rise | Unable without assistance or multiple attempts = 0 |  |
|  | Able but requires more than one attempt = 1 |  |
|  | Able to rise with one attempt = 2 |  |
| 4. Immediate Balance After Standing (first 5 seconds) | Unsteady (sways, moves feet, marked trunk sway) = 0 |  |
|  | Steady, but requires use of walker, cane, or other support = 1 |  |
|  | Steady without walker, cane, or other support = 2 |  |
| 5. Standing Balance | Unsteady = 0 |  |
|  | Steady but feet apart, or requires support = 1 |  |
|  | Steady, narrow base without support = 2 |  |
| 6. Nudging (patient is pushed lightly on sternum with light nudges 3 times) | Starts to fall = 0 |  |
|  | Sways, grips chair or self = 1 |  |
|  | Steady = 2 |  |
| 7. Eyes Closed (same position for 6) | Unsteady = 0 |  |
|  | Steady = 1 |  |
| 8. Turn 360° | Steps discontinuous = 0 |  |
|  | Steps continuous = 1 |  |
|  | Unsteady while turning (grabbing, swaying) = 0 |  |
|  | Steady while turning = 1 |  |
| 9. Sitting Down | Unsafe (misjudged distance, falls into chair) = 0 |  |
|  | Uses arms or moves awkwardly = 1 |  |
|  | Safe, smooth motion = 2 |  |
| **Balance Test Score:** | | |
| **Part Two: Gait Test**  **(The patient walks back and forth for a distance of approximately 3 meters, initially at a normal pace and then returns at a faster but safe pace.)** | | |
| **Evaluation Item** | **Evaluation Criteria** | **Score** |
| 1. Initiation of Gait | Any hesitancy or multiple attempts to start = 0 |  |
|  | Initiates gait without hesitation = 1 |  |
| 2. Step Length and Height |  |  |
| a. Left Foot Clearance | Foot drags or does not clear by more than 2.5–5 cm = 0 |  |
|  | Foot clears floor but does not exceed 2.5–5 cm = 1 |  |
| b. Right Foot Clearance | Foot drags or does not clear by more than 2.5–5 cm = 0 |  |
|  | Foot clears floor but does not exceed 2.5–5 cm = 1 |  |
| c. Left Foot Step Length | Does not step past the opposite stance foot = 0 |  |
|  | Steps past the opposite stance foot = 1 |  |
| d. Right Foot Step Length | Does not step past the opposite stance foot = 0 |  |
|  | Steps past the opposite stance foot = 1 |  |
| 3. Step Symmetry | Steps are unequal = 0 |  |
|  | Steps are equal = 1 |  |
| 4. Step Continuity | Stopping between steps or discontinuity of step rhythm = 0 |  |
|  | Steps appear continuous = 1 |  |
| 5. Path Deviation (walks approximately 3 meters) | Marked deviation to one side = 0 |  |
|  | Mild to moderate deviation or use of walking aid = 1 |  |
|  | Walks straight without deviation or aid = 2 |  |
| 6. Trunk Stability | Marked sway or uses assistive device = 0 |  |
|  | No sway, but knees or trunk flexion is evident while maintaining balance = 1 |  |
|  | No sway, no knee or trunk flexion, and no assistive device used = 2 |  |
| 7. Foot Clearance (Width of Foot Separation) | Heels apart (stride width ≥ 0) = 0 |  |
|  | Heels nearly touching while walking = 1 |  |
| **Gait Test Score:** | | |
| **Total Score:**  The total score is the sum of the scores from both sections, with a higher score indicating better mobility and balance ability. A total score >24 suggests no fall risk, a score between 19-24 indicates a risk of falls, and a score <19 suggests a high risk of falls. | | |
