# Supplementary material for: Surface electromyographic characteristics of lower limb muscles in frail older adults: Protocol for an observational case - control study
Source: PLoS One. 2025 Jul 3;20(7):e0325356. doi: 10.1371/journal.pone.0325356 (PMC12225879; doi:10.1371/journal.pone.0325356)
Supplement: Peer review history

# Editor report: Decision Letter 0

**DOI:** 10.1371/journal.pone.0325356.r001

**Authors:** Jennifer Tucker

Surface electromyographic characteristics of lower limb muscles in frail older adults: protocol for an observational case - control study

PONE-D-25-15575

Dear Dr. Chen,

We’re pleased to inform you that your manuscript has been judged scientifically suitable for publication and will be formally accepted for publication once it meets all outstanding technical requirements.

Within one week, you’ll receive an e-mail detailing the required amendments. When these have been addressed, you’ll receive a formal acceptance letter and your manuscript will be scheduled for publication.

An invoice will be generated when your article is formally accepted. Please note, if your institution has a publishing partnership with PLOS and your article meets the relevant criteria, all or part of your publication costs will be covered. Please make sure your user information is up-to-date by logging into Editorial Manager at Editorial Manager®  and clicking the ‘Update My Information' link at the top of the page. If you have any questions relating to publication charges, please contact our Author Billing department directly at authorbilling@plos.org.

If your institution or institutions have a press office, please notify them about your upcoming paper to help maximize its impact. If they’ll be preparing press materials, please inform our press team as soon as possible -- no later than 48 hours after receiving the formal acceptance. Your manuscript will remain under strict press embargo until 2 pm Eastern Time on the date of publication. For more information, please contact onepress@plos.org.

Kind regards,

Jennifer Tucker, PhD

Staff Editor

PLOS ONE

Additional Editor Comments (optional):

We are pleased to accept your study protocol for publication in PLOS One. 

# Editor report: Acceptance letter

**DOI:** 10.1371/journal.pone.0325356.r002

**Authors:** Jennifer Tucker

PONE-D-25-15575

PLOS ONE

Dear Dr. Chen,

I'm pleased to inform you that your manuscript has been deemed suitable for publication in PLOS ONE. Congratulations! Your manuscript is now being handed over to our production team.

At this stage, our production department will prepare your paper for publication. This includes ensuring the following:

* All references, tables, and figures are properly cited

* All relevant supporting information is included in the manuscript submission,

* There are no issues that prevent the paper from being properly typeset

You will receive further instructions from the production team, including instructions on how to review your proof when it is ready. Please keep in mind that we are working through a large volume of accepted articles, so please give us a few days to review your paper and let you know the next and final steps.

Lastly, if your institution or institutions have a press office, please let them know about your upcoming paper now to help maximize its impact. If they'll be preparing press materials, please inform our press team within the next 48 hours. Your manuscript will remain under strict press embargo until 2 pm Eastern Time on the date of publication. For more information, please contact onepress@plos.org.

If we can help with anything else, please email us at customercare@plos.org.

Thank you for submitting your work to PLOS ONE and supporting open access.

Kind regards,

PLOS ONE Editorial Office Staff

on behalf of

Dr Jennifer Tucker

Staff Editor

PLOS ONE

Enable Ginger *Cannot connect to Ginger* Check your internet connectionor reload the browser Disable Ginger Rephrase Rephrase with Ginger (Ctrl+Alt+E) 5 Log in to edit with Ginger Ginger is checking your text for mistakes... Disable Ginger in this text field Disable Ginger on this website 600/41967 free characters checked. Go Premium to check longer texts and entire documents
